# Adipocyte in vascular wall can induce the rupture of abdominal aortic aneurysm

**DOI:** 10.1038/srep31268

**Published:** 2016-08-08

**Authors:** Hirona Kugo, Nobuhiro Zaima, Hiroki Tanaka, Youhei Mouri, Kenichi Yanagimoto, Kohsuke Hayamizu, Keisuke Hashimoto, Takeshi Sasaki, Masaki Sano, Tatsuro Yata, Tetsumei Urano, Mitsutoshi Setou, Naoki Unno, Tatsuya Moriyama

**Affiliations:** 1 Department of Applied Biological Chemistry, Graduate School of Agriculture, Kindai University, 204-3327 Nakamachi, Nara City, Nara 631-8505, Japan; 2 Department of Medical Physiology, Hamamatsu University School of Medicine, Shizuoka, Japan; 3 Human Life Science R&D Center, Nippon Suisan Kaisha, Ltd., Tokyo, Japan; 4 General Health Medical Center, Yokohama University of Pharmacy, Japan; 5 Department of Anatomy and Neuroscience, Hamamatsu University School of Medicine, Japan; 6 Division of Vascular Surgery, Second Department of Surgery, Hamamatsu University School of Medicine, Japan; 7 International Mass Imaging Center Department of Cellular and Molecular Anatomy, and Preeminent Medical Photonics Education & Research Center Department of Systems Molecular Anatomy, Hamamatsu University School of Medicine, 1-20-1 Handayama, Higashi-ku, Hamamatsu, Shizuoka 431-3192, Japan; 8 Department of Anatomy, The university of Hong Kong, 6/F, William MW Mong Block 21 Sassoon Road, Pokfulam, Hong Kong SAR, China; 9 Division of Neural Systematics, National Institute for Physiological Sciences, 38 Nishigonaka Myodaiji, Okazaki, Aichi, 444-8585, Japan

## Abstract

Abdominal aortic aneurysm (AAA) is a vascular disease involving the gradual dilation of the abdominal aorta. It has been reported that development of AAA is associated with inflammation of the vascular wall; however, the mechanism of AAA rupture is not fully understood. In this study, we investigated the mechanism underlying AAA rupture using a hypoperfusion-induced animal model. We found that the administration of triolein increased the AAA rupture rate in the animal model and that the number of adipocytes was increased in ruptured vascular walls compared to non-ruptured walls. In the ruptured group, macrophage infiltration and the protein levels of matrix metalloproteinases 2 and 9 were increased in the areas around adipocytes, while collagen-positive areas were decreased in the areas with adipocytes compared to those without adipocytes. The administration of fish oil, which suppresses adipocyte hypertrophy, decreased the number and size of adipocytes, as well as decreased the risk of AAA rupture ratio by 0.23 compared to the triolein administered group. In human AAA samples, the amount of triglyceride in the adventitia was correlated with the diameter of the AAA. These results suggest that AAA rupture is related to the abnormal appearance of adipocytes in the vascular wall.

Abdominal aortic aneurysm (AAA) is a disease that involves the progressive dilation of the abdominal aorta. The risk factors associated with AAA have been reported to include age, sex, smoking status, and hypertension^1^. Although the detailed molecular mechanism of AAA development is not fully understood, it has been established that AAA is closely associated with weakening of the vascular wall due to inflammation^2^. AAA is histologically characterized by oxidative stress, chronic inflammation, and the degradation of extracellular matrix^3^. Inflammatory cells, such as neutrophils, monocytes, and macrophages, and inflammatory cytokines, such as monocyte chemoattractant protein (MCP)-1, tumor necrosis factor (TNF)-α, and interleukin (IL)-6, are increased in the aortic wall of AAA^4^,^5^. Subsequently, increased matrix metalloproteinase (MMP) proteins disrupt the elastin and collagen fibers that play an important role in maintaining the integrity and elasticity of the vascular wall^6^. It has been reported that the activation of MMP2 and MMP9 is especially associated with human aneurysm formation^6^,^7^.

Increased aneurysm diameter is a significant risk factor for AAA rupture^8^. Patients with increased risk of rupture have no choice but to undergo surgery by means of either open repair with prosthetic graft replacement or endovascular stent graft placement^3^. Clinically, it is usual to measure aneurysmal diameter periodically in the outpatient clinic until the size reaches 55 mm, at which point surgeons recommend that the patient undergo surgery^8^. The decision to perform AAA surgery is derived from the balance between the patient’s operative risk and the risk of aneurysm rupture. Currently, there is no effective medicine available for inhibiting aneurysm growth or for preventing aneurysm rupture; this can be attributed to the undefined mechanisms of AAA development.

We recently reported that hypoperfusion of the vascular wall occurred in human AAA tissue due to adventitial vasa vasorum arteriosclerosis^9^. Next, using a hypoperfusion-induced animal model, we demonstrated that hypoperfusion of the vascular wall caused AAA development^10^. In preliminary study, we observed not only aortic dilation, but also spontaneous AAA rupture, in the AAA model. However, the rate of AAA rupture was too low (around 15%) to obtain enough samples for pathological analysis of the ruptured vascular wall. In the current study, we found that the administration of triolein, a triglyceride (TG) species, increased the AAA rupture rate in the animal model. We then performed pathological analysis of the ruptured vascular walls to clarify the mechanism of AAA rupture.

## Results

### Effect of triolein administration on AAA rupture

**Figure 1:**
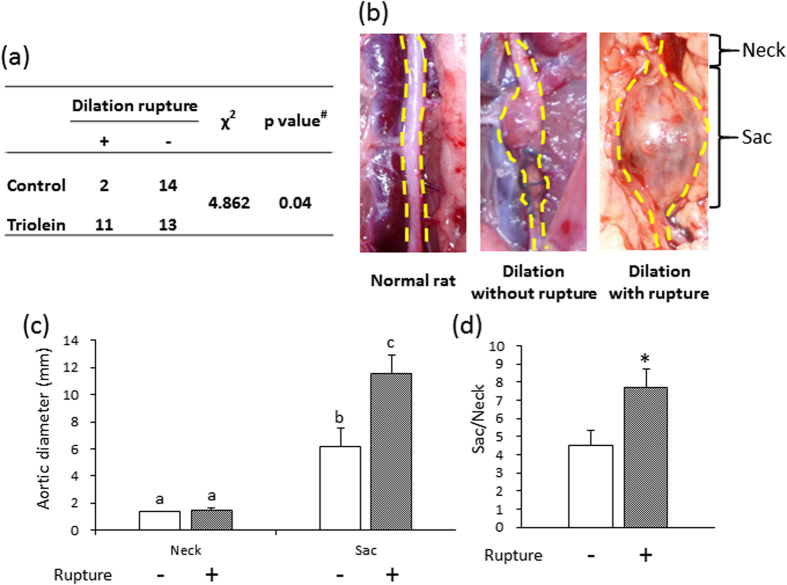
The effects of triolein administration on AAA rupture and aortic diameter. (**a**) The effect of triolein administration on AAA rupture ratio. Data are presented as number of rats. ^#^P value of Fisher’s exact test. (**b**) Representative images of the abdominal aorta from a normal rat and from rats in the triolein-administered group. The ruptured AAA is shown after washing away blood. (**c**) Quantitative analysis of aortic diameter. (**d**) Dilation ratio of the non-ruptured and ruptured groups. Data are the mean ± s.e.m. Non-ruptured (n = 13), ruptured (n = 11). *P < 0.01 versus non-ruptured group.

After treatment, to induce hypoperfusion of the vascular wall, rats were divided into two groups: a control group, administered water; and a test group, administered triolein. The effect of triolein administration on AAA rupture is shown in Fig. 1a. Triolein significantly increase the AAA rupture risk ratio by 3.67 compared to the control group (*P* < 0.05). To clarify the cause of the rupture, we further divided the triolein group into a non-ruptured group and a ruptured group. We observed an area with a non-dilated diameter (neck) in both groups, and this aortic diameter was equal to the aortic diameter in normal rats without any treatment (Fig. 1b). The neck area was set as control for each group because fibers in this area were not different from those in the untreated normal vascular wall^10^. A ruptured AAA, pictured before washing away the blood, is shown in Supplementary Fig. S1. The diameter of the dilated aorta (sac) in the ruptured group was significantly increased compared with that in the non-ruptured group (Fig. 1c). The dilation ratio (sac/neck) was also significantly increased in the ruptured group (Fig. 1d). The initial body weight (g) and the gain in body weight (g) were not significantly different between the ruptured group and the non-ruptured group (Supplementary Fig. S2a,b). Serum TG levels and total cholesterol levels were also not significantly different between the two groups (Supplementary Fig. S2c,d). Pathological features other than AAA, such as sepsis or rapid weight loss, were not observed in either the ruptured group or the non-ruptured group (data not shown).

### Thickness of vascular wall, elastin degradation score, and thickness of medial wall

The vascular wall of the AAA sac was significantly thickened in both ruptured and non-ruptured groups compared to the vascular wall of the AAA neck in each group (Supplementary Fig. S3a–d,i). The AAA sac vascular wall in the ruptured group was thicker than that in the non-ruptured group (Supplementary Fig. S3i). Elastin fibers in the vascular wall were observed by Elastica van Gieson (EVG) staining (Supplementary Fig. S3e–h). The elastin degradation score of the ruptured group was not significantly different to that of the non-ruptured group (Supplementary Fig. S3j). The area of smooth muscle cells of the AAA sac was significantly decreased compared with that of neck. (Supplementary Fig. S4a–e). The medial wall of the AAA sac was significantly thinner than that of the AAA neck in both groups (Supplementary Fig. S4f). However, there was no difference between the thickness of the medial wall in the ruptured group and the non-ruptured group (Supplementary Fig. S4f).

### Adipocytes in the vascular wall

**Figure 2:**
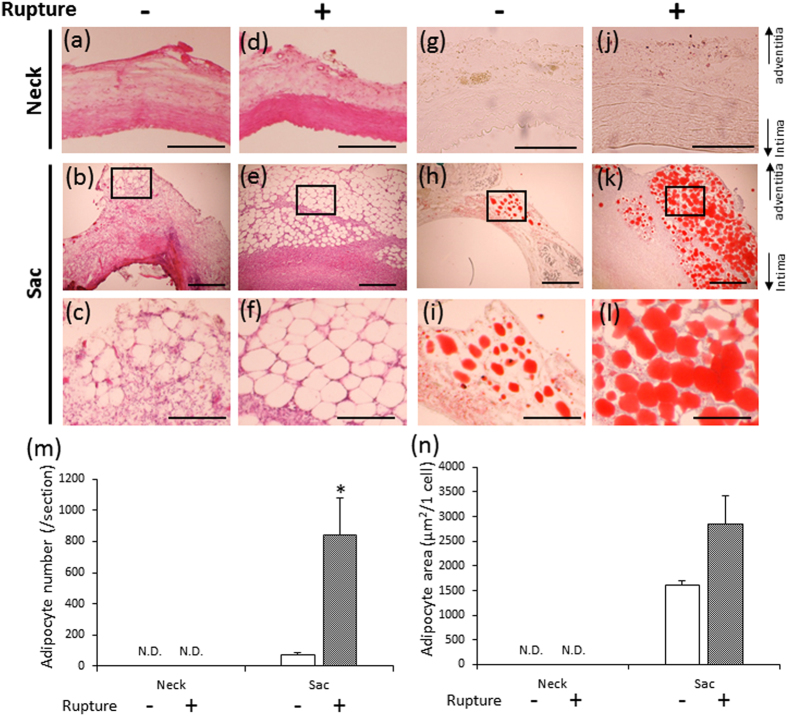
Quantification of adipocytes in non-ruptured and ruptured groups. (**a**–**f**) Representative images of hematoxylin-eosin (HE) staining (**a,c,d,f**: scale bar = 200 μm; **b**,**e**: scale bar = 500 μm). (**g**–**l**) Representative images of Oil Red O staining (**g**,**i**: scale bar = 100 μm; **h**,**k**: scale bar = 500 μm; **i**,**l**: scale bar = 200 μm). The square area in the middle panels is magnified in the bottom panels as a representative image. (**m**) Quantification of adipocyte number in the non-ruptured and ruptured groups. (**n**) Quantification of adipocyte area in the non-ruptured and ruptured groups. Data are the mean ± s.e.m. Non-ruptured (n = 7), ruptured (n = 8). *P < 0.01 versus non-ruptured group. N.D. = not detected.

Adipocyte-like cells were observed in the vascular adventitial of the AAA sac, but not in the neck vascular wall, for both ruptured and non-ruptured groups (Fig. 2a–f). Electron microscopy showed that these cells possessed the characteristic morphology of adipocytes (Supplementary Fig. S5b). In addition, peroxisome proliferator-activated receptor γ (PPARγ) (Supplementary Fig. S5f) was detected in the nucleus of these cells. These regions of adipocyte-like cells also stained positively with Oil Red O (Fig. 2g–l). Taken together, these data indicate that the observed cells were adipocytes. The number of adipocytes in the vascular wall of the AAA sac in the ruptured group was significantly increased compared to the non-ruptured group (Fig. 2m). The difference in adipocyte size (μm^2^/cell) between groups was not statistically significant (Fig. 2n).

### Collagen fibers and MMP expression

**Figure 3:**
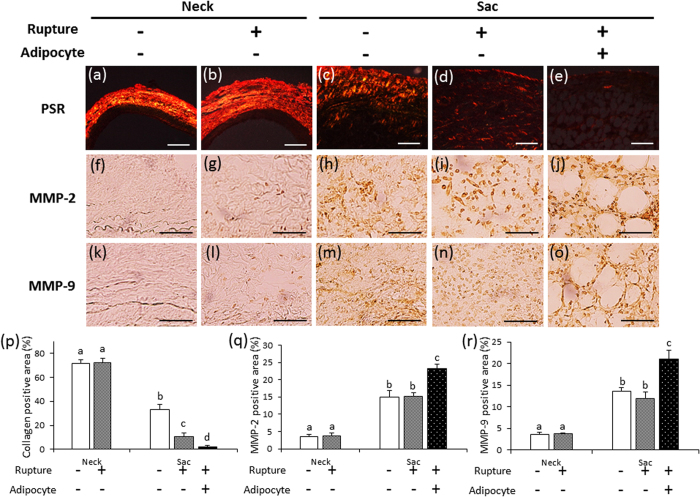
PSR staining and immunohistochemical staining for MMP2 and MMP9. (**a**–**e**) Representative images of PSR staining (scale bar = 200 μm). (**f**–**j**) Representative images of immunostaining for MMP2 (scale bar = 50 μm). (**k**–**o**) Representative images of immunostaining for MMP9 (scale bar = 50 μm). (**p**) Quantification of the collagen-positive area of the vascular wall. (**q**) Quantification of MMP2-positive areas of the vascular wall. (**r**) Quantification of MMP9-positive areas of the vascular wall. Data are the mean ± s.e.m. Non-ruptured (n = 3), ruptured (n = 5). Values with different letters are significantly different (*P* < 0.05).

Since it has been speculated that the abnormal appearance of adipocytes in the vascular wall is associated with AAA rupture (Fig. 2), we divided the ruptured AAA tissues into two groups: those with adipocytes and those without adipocytes. Collagen fibers were observed using PicroSirius Red (PSR) staining (Fig. 3a–e). The collagen-positive area was significantly decreased in the sac vascular wall, compared with the neck vascular wall, of each group (Fig. 3p). The collagen-positive area in the ruptured wall was significantly decreased compared to the non-ruptured wall (Fig. 3p). In addition, the density of collagen fibers was significantly decreased in the area with adipocytes, compared to the area without adipocytes, in the ruptured vascular wall (Fig. 3p).

Immunohistochemical examination showed the expression of MMP2 and MMP9 in each group (Fig. 3f–o). Areas positive for MMP2 and MMP9 were significantly greater in the sac vascular wall than in the neck vascular wall of each group (Fig. 3q,r). There was no significant difference in MMP2- and MMP9-positive areas between the non-ruptured sac vascular wall and the area without adipocytes in the ruptured vascular wall (Fig. 3q,r). On the contrary, MMP2- and MMP9-positive areas were significantly greater in the areas with adipocytes than in the areas without adipocytes in the ruptured vascular wall (Fig. 3q,r).

### MCP-1 and MAC387^+^ monocytes/macrophages in the vascular wall

**Figure 4:**
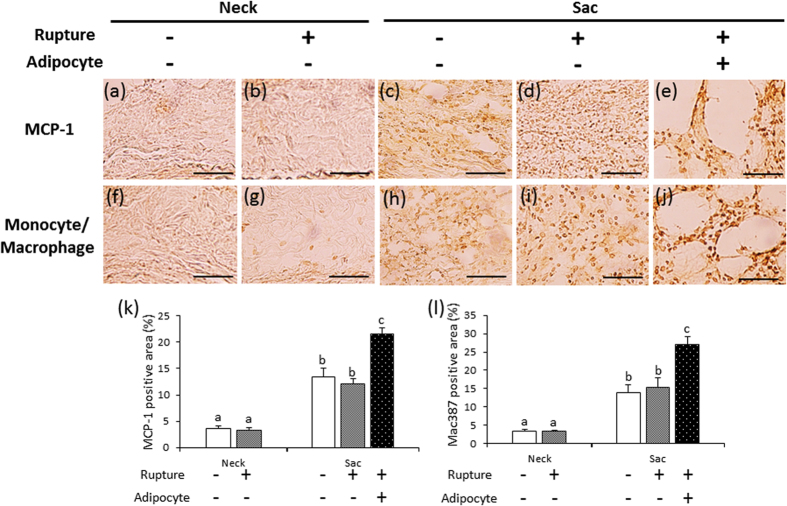
Immunohistochemical staining for MCP-1 and MAC387^+^ monocytes/macrophages. (**a–e**) Representative images of immunostaining for MCP-1 (scale bar = 50 μm). (**f–j**) Representative images of immunostaining for MAC387^+^ monocytes/macrophages (scale bar = 50 μm). (**k**) Quantification of MCP-1-positive areas of the vascular wall. (**l**) Quantification of MAC387^+^ monocyte/macrophage-positive areas of the vascular wall. Data are the mean ± s.e.m. Non-ruptured (n = 3), ruptured (n = 5). Values with different letters are significantly different (*P* < 0.05).

To investigate the mechanism underlying the increased protein levels of MMP2 and MMP9 and the decreased collagen in the areas with adipocytes, we performed immunostaining for MCP-1, MAC387^+^ monocytes/macrophages (potentially M1-like macrophages) (Fig. 4a–j), and CD163^+^ macrophages (consistent with M2-like macrophages) (Supplementary Fig. S6a–e). The areas positive for MCP-1 were significantly greater in the sac vascular wall, compared to the neck vascular wall, of each group (Fig. 4k). MCP-1-positive areas were significantly greater in the areas with adipocytes in the ruptured sac vascular walls, compared to the non-ruptured vascular walls and the areas without adipocytes in the ruptured vascular walls (Fig. 4k). MAC387^+^ monocytes/macrophages-positive areas were significantly greater in the sac vascular wall, compared to the neck vascular wall, of each group (Fig. 4l). MAC387^+^ monocytes/macrophages-positive areas were significantly greater in the areas with adipocytes in the ruptured sac vascular walls, compared to the non-ruptured vascular walls and the areas without adipocytes in the ruptured vascular walls (Fig. 4l). CD163^+^ macrophages-positive areas were significantly greater in the sac vascular wall, compared to the neck vascular wall, of each group (Supplementary Fig. S6f). CD163^+^ macrophages-positive areas were significantly greater in the areas with adipocytes in the ruptured sac vascular walls, compared to the non-ruptured vascular walls and the areas without adipocytes in the ruptured vascular walls (Supplementary Fig. S6f). These data suggest that local inflammation is associated with adipocytes.

### Effect of fish oil, another kind of TG, on AAA rupture

Since it has been speculated that the abnormal appearance adipocytes induced by the administration of triolein is associated with AAA rupture (Figs 3 and 4), we next compared the effects of triolein with fish oil, another kind of TG, which suppresses growth of adipocyte^11^. The average final body weight (g) of the rats was not significantly different between the three groups (343.9 ± 44.0 g in the control group, 341.6 ± 29.0 g in the triolein group, and 373.7 ± 20.4 g in the fish oil group; Supplementary Fig. S7a). Serum TG levels in the fish oil group were significantly decreased compared with the triolein group (Supplementary Fig. S7b). Serum total cholesterol levels were not significantly different between the three groups (Supplementary Fig. S7c).

**Figure 5:**
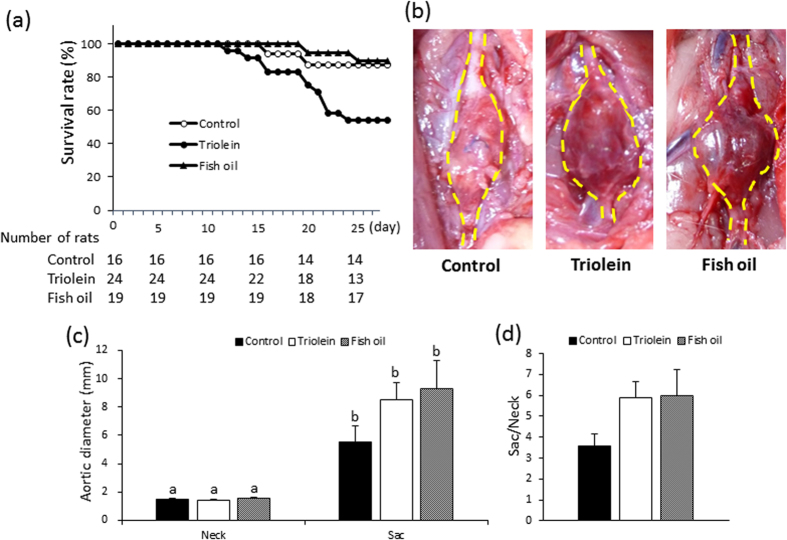
Suppressive effects of fish oil on AAA rupture. (**a**) Kaplan-Meier curves for AAA rupture. (**b**) Representative images of abdominal aorta. (**c**) Quantitative analysis of aortic diameter. (**d**) Dilation ratio of the triolein and fish oil groups. Data are the mean ± s.e.m. Control group (n = 16), triolein group (n = 24), fish oil group (n = 19).

The Kaplan–Meier curves for AAA rupture are shown in Fig. 5a. The AAA rupture risk in fish oil administrated group significantly decreased compared to triolein administrated group (p = 0.0348). The probability of AAA rupture in the control group was not significantly different from either the triolein group (p = 0.0734) or the fish oil group (*P* = 0.821). The aneurysm formation observed for each of the three groups is shown in Fig. 5b. AAA sac diameter and the dilation ratio (sac/neck) were not significantly different between the groups (Fig. 5c,d).

### Vascular pathology of the fish oil group

**Figure 6:**
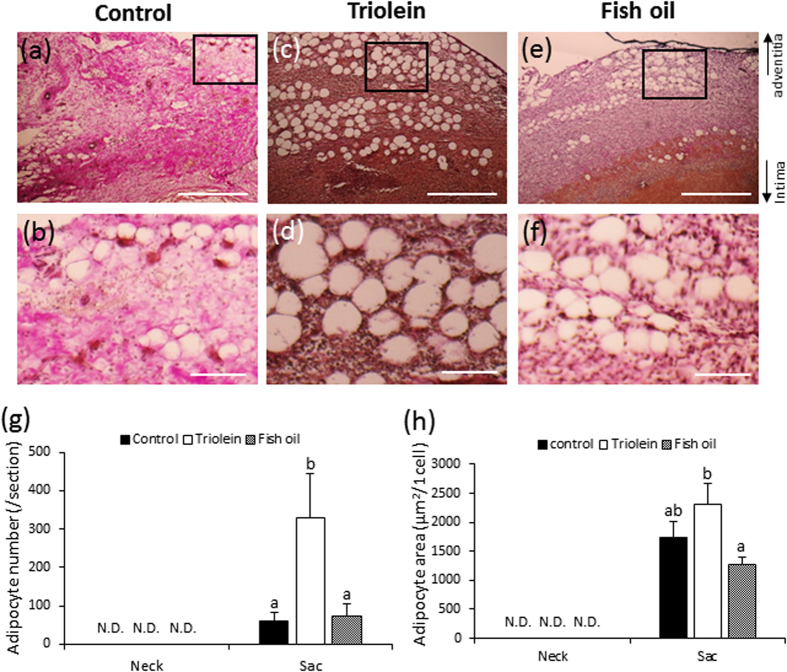
Quantification of adipocytes in the triolein and fish oil groups. (**a–f**) Representative images of the vascular wall where adipocytes were observed (**a,c,e:** scale bar = 500 μm; **b,d,f:** scale bar = 100 μm). The square area in the upper panels is magnified in the bottom panels as a representative image. (**g**) Quantification of adipocyte number in the control, triolein or fish oil groups. (**h**) Quantification of adipocyte area in the control, triolein or fish oil groups. Data are the mean ± s.e.m. Control group (n = 14), triolein group (n = 15), fish oil group (n = 14). Values with different letters are significantly different (*P* < 0.05). N.D. = not detected.

Adipocytes were observed in the AAA sac wall for all groups (Fig. 6a–f). The number (/section) and size (μm^2^/cell) of adipocytes were significantly decreased in the fish oil group compared to the triolein group (Fig. 6g,h). The number and size of adipocytes in the fish oil group were not significantly different from those in the control group (Fig. 6g,h).

**Figure 7:**
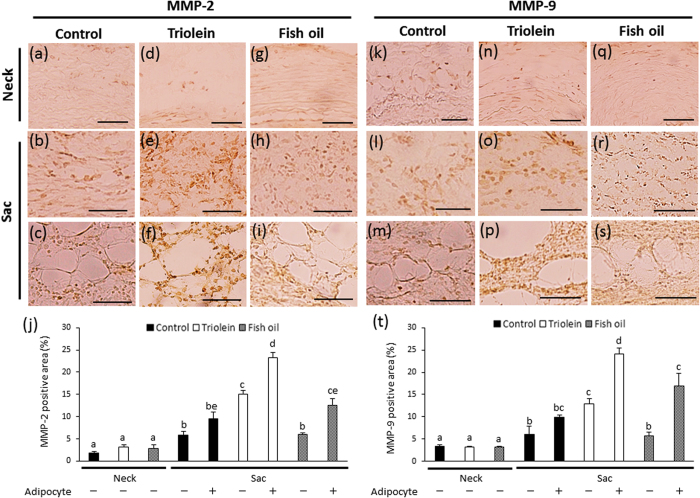
Immunohistochemical staining for MMP2 and MMP9. AAA sac areas from the three experimental groups were divided into two groups: those without adipocytes (−) (**b**,**e**,**h**,**l**,**o**,**r**) and those with adipocytes (+) (**c**,**f**,**i**,**m**,**p**,**s**). (**a**–**i**) Representative images of immunostaining for MMP2 (scale bar = 50 μm). (**j**) Quantification of MMP2-positive areas of the vascular wall. (**k–s**) Representative images of immunostaining for MMP9 (scale bar = 50 μm). (**t**) Quantification of MMP9-positive areas of the vascular wall. Data are the mean ± s.e.m. Control group (n = 9), triolein group (n = 10), fish oil group (n = 8). Values with different letters are significantly different (*P* < 0.05).

For histological analysis, AAA sac areas from the three experimental groups were divided into two groups: those with adipocytes and those without adipocytes. The thickness of the aortic wall was not significantly different between the three groups (Supplementary Fig. S8). The elastin degradation score was also not significantly different between the three groups (Supplementary Fig. S9a–j). In the triolein group, the collagen-positive areas were significantly decreased in the AAA sac walls with adipocytes compared to those without adipocytes (Supplementary Fig. S9k–t). In the fish oil group, the collagen-positive areas were not significantly different in the AAA sac walls compared to those without adipocytes (Supplementary Fig. S9k–t). The smooth muscle-positive areas were not different between the three groups (Supplementary Fig. S10a–g). The thickness of the medial wall in the AAA neck and sac was also not different between the three groups (Supplementary Fig. S10h). Areas positive for MMP2 and MMP9 in the AAA sac walls with adipocytes were significantly decreased in the fish oil group compared to the triolein group (Fig. 7). Areas positive for MMP2 and MMP9 were not significantly different between the control and fish oil groups (Fig. 7). Areas positive for MCP-1 in the AAA sac walls with adipocytes were significantly decreased in the fish oil group compared to the triolein group (Supplementary Fig. S11a–j). Areas positive for macrophages in the AAA sac walls with adipocytes were also significantly decreased in the fish oil group compared to the triolein group (Supplementary Fig. S11k–t). Areas positive for MCP-1 and MAC387^+^ monocytes/macrophages in the AAA sac walls with adipocytes were not significantly different between the control and fish oil groups (Supplementary Fig. S11j,t).

### Correlation between the amount of TG in human AAA vascular walls and AAA diameter

**Figure 8:**
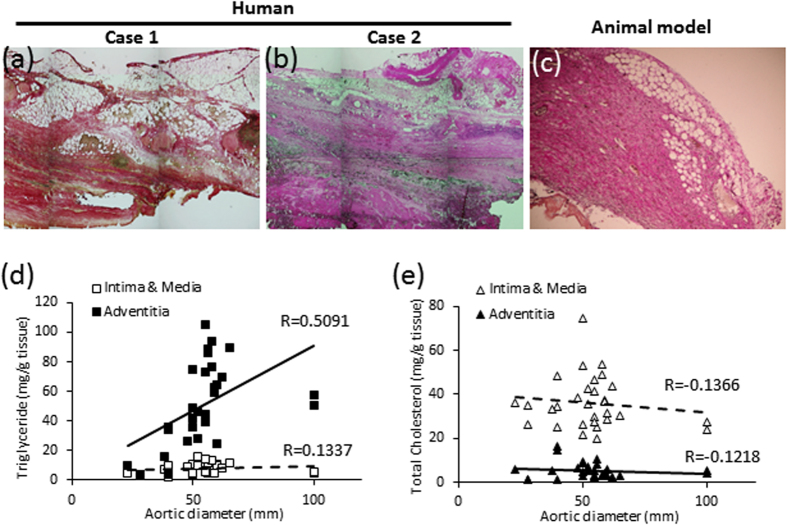
Adipocytes in the vascular wall in both human samples and animal models and the correlation between lipids in the aorta and the aortic diameter of human AAA samples. Adipocytes were observed in the AAA vascular wall in human samples (**a**,**b**) as well as in animal models (**c**). The abnormal appearance of adipocytes in human AAA vascular wall has been previously reported^12^. (**d**) The triglyceride amount in aortas versus aortic diameter (mm). (**e**) The total cholesterol amount in aortas versus aortic diameter (mm). Aortas were divided into two groups: 1) intima and media, and 2) adventitia.

Since the results of the animal studies strongly suggested that the abnormal appearance of adipocytes in the vascular wall is related to AAA rupture, we investigated the relationship between the TG content in human AAA vascular walls and AAA diameter. As the abnormal appearance of adipocytes was mainly observed in the adventitia in both humans and the AAA animal model (Fig. 8a–c), we divided the human AAA vascular wall into two groups: 1) the intima and media, and 2) the adventitia. The amount of TG in the adventitia was correlated with AAA diameter (Fig. 8d; R = 0.5091). On the contrary, the amount of TG in the intima and media was not correlated with AAA diameter (Fig. 8d; R = 0.1337). Total cholesterol in the vascular wall was not correlated with AAA diameter (Fig. 8e; R = −0.1366 in the intima and media, and R = −0.1218 in the adventitia). The amount of TG in the vascular wall was not correlated with serum TG levels (Supplementary Fig. S12a), serum total cholesterol levels (Supplementary Fig. S12b), or body mass index (BMI) (Supplementary Fig. S12c). Similarly, the amount of cholesterol in the vascular wall was not correlated with serum TG levels (Supplementary Fig. S12d), serum total cholesterol levels (Supplementary Fig. S12e), or BMI (Supplementary Fig. S12f). The aortic diameter of AAA patients was also not correlated with serum TG levels (Supplementary Fig. S12g), serum total cholesterol levels (Supplementary Fig. S12h), or BMI (Supplementary Fig. S12i).

## Discussion

We observed spontaneous AAA rupture in a hypoperfusion-induced animal model that we previously established^10^. However, the incidence rate of AAA rupture was too low (around 15%) to obtain enough samples for pathological analysis. In this study, we found that the administration of triolein, a kind of TG, increased rupture rates in the experimental model, which enabled us to assess the difference between ruptured and non-ruptured AAA (Fig. 1). Therefore, we used triolein administration in the animal model to clarify the mechanism of AAA rupture. The number of adventitial adipocytes was significantly increased in the ruptured vascular wall compared to the non-ruptured vascular wall (Fig. 2). MMP2 and MMP9 protein levels around the region with adipocytes were significantly increased compared to levels around the region without adipocytes (Fig. 3). The collagen-positive area was significantly decreased around the region with adipocytes (Fig. 3). MCP-1 and macrophages were significantly increased around adipocytes (Fig. 4 and Supplementary Fig. S6). The AAA rupture rate in fish oil (other kind of TG, which suppresses growth of adipocyte)^11^ group significantly decreased by 0.23 compared to triolein administrated group (Fig. 5). The number and size of adipocytes and the protein levels of MMP2 and MMP9 were all decreased in the fish oil group compared to the triolein group (Figs 6 and 7). The thickness of the medial wall in the AAA neck and sac was not different between the control, triolein, and fish oil groups (Supplementary Fig. S10h). These data strongly suggest that the abnormal appearance of adipocytes in the vascular wall is involved in AAA rupture.

In this study, the triolein group was divided into two groups: non-ruptured and ruptured (Fig. 1). The aortic diameters in the ruptured group were increased compared to those in the non-ruptured group (Fig. 1c). The area of smooth muscle cells of the AAA sac was significantly decreased compared with that of neck. (Supplementary Fig. S4a–e). The thickness of the medial wall in the AAA sac was significantly thinner than that in the AAA neck (Supplementary Fig. S4f). On the contrary, there was no difference between the thickness of the medial wall in ruptured cases and non-ruptured cases (Supplementary Fig. S4f). These data suggest that the depletion of medial smooth muscle cells was associated with the formation of AAA but not with AAA rupture in our experimental model. Adipocytes were observed in the sac area, but not in the neck area, of both ruptured and non-ruptured groups (Fig. 2). This abnormal appearance of adipocytes is consistent with our previous reports in human AAA tissue^12^. The number of adipocytes in the sac area of the ruptured group was significantly increased compared to that of the non-ruptured group (Fig. 2m). Pathological analysis showed that the destruction of collagen fibers and an increase in MMP2 and MMP9 protein levels were especially prominent in the areas around adipocytes (Fig. 3). In addition, increased MCP-1 protein levels and the infiltration of macrophages were observed around the adipocytes (Fig. 4). These results suggest that the inflammation around adipocytes was induced by the increased hypertrophic adipocytes^13^. Specifically, the increase in hypertrophic adipocytes enhances MCP-1 expression, which leads to macrophage infiltration. The increased inflammatory hypertrophic adipocytes and recruited macrophages could cause the increase in MMP2 and MMP9 and the destruction of collagen fibers around adipocytes. In addition to the potential induction of inflammation around adipocytes, electron microscopy showed that the very existence of adipocytes caused decreased collagen in the vascular wall (Supplementary Fig. S5a,b). Taken together, these results indicate that the integrity of the vascular wall in the area around adipocytes is decreased compared to that in the area without adipocytes.

We investigated the effect of fish oil on the incidence rate of rupture because fish oil has an inhibitory effect on the growth of adipocytes^11^. The risk of AAA rupture in the fish oil group was significantly decreased compared to the triolein group (Fig. 5). The number and size of adipocytes in the fish oil group were significantly decreased compared to the triolein group, indicating that fish oil suppresses adipocyte hypertrophy in the vascular wall of this animal model (Fig. 6). In the triolein group, the collagen-positive area in the AAA sac wall with adipocytes was significantly decreased compared to the area without adipocytes (Supplementary Fig. S9t). By contrast, in the fish oil group, the collagen-positive area in the AAA sac wall with adipocytes was not significantly different to the area without adipocytes (Supplementary Fig. S9t). Protein levels of MMP2, MMP9, MCP1, and macrophages around the adipocytes were significantly decreased in the fish oil group compared to the triolein group (Fig. 7 and Supplementary Fig. S11). Since it has been established that adipocyte hypertrophy can cause chronic inflammation^13^, the suppression of inflammation around adipocytes in the vascular wall of the fish oil group can be attributed to the decreased number and size of adipocytes. These results reveal the possibility that AAA rupture may be prevented by the appropriate control of adipocytes in the vascular wall.

The fish oil used in this study contained 30.8% eicosapentaenoic acid and 15.7% docosahexaenoic acid as the major fatty acid components. These n-3 polyunsaturated fatty acids (PUFAs) suppress de novo TG synthesis via inhibition of the expression of sterol regulatory element-binding protein 1c because they are antagonists of liver X receptor α^14^. The suppression of adipocyte hypertrophy can be attributed to the inhibitory effect on TG synthesis of the n-3 PUFAs in fish oil. The lower serum levels of TG in the fish oil group may be partly due to the suppression of adipocyte hypertrophy (Supplementary Fig. S7b). In addition to this suppressive effect on adipocyte hypertrophy, it is possible that the anti-inflammatory effect of the n-3 PUFAs in fish oil also contributed to the suppression of adipocyte-induced inflammation.

There was a positive correlation between the amount of TG in the adventitia of human AAA vascular walls and AAA diameter (Fig. 8). Since we recently reported increased adipocytes in the adventitia of human AAA vascular walls^12^, this increased TG reflects the increased number and/or hypertrophy of adipocytes in the vascular wall. The results of this study strongly suggest that the abnormal appearance of adipocytes in the AAA vascular wall is related to AAA rupture in humans as well as in animal models. In case-control studies, the risk of death from AAA rupture is correlated with serum TG levels^15^,^16^. A high serum TG level may induce adipocyte hypertrophy in human vascular walls. In both humans and in the animal model, hypoperfusion of the vascular wall is likely to trigger the abnormal appearance of adipocytes^9^. However, the mechanisms underlying the abnormal appearance of adipocytes remain unknown and further studies are needed.

In this study, we demonstrated the relationship between the abnormal appearance of adipocytes in the vascular wall and AAA rupture by comparing the effects of two different TG species (triolein and fish oil) on the risk of AAA rupture. Our results suggest that the appropriate control of vascular adipocytes may treat or prevent AAA rupture. Fish oil or n-3 PUFA-containing drugs or foods may therefore be effective for the prevention of AAA rupture. There are some limitations to our study that deserve discussion. Time-dependent increases of the aortic diameters were not assessed for this study. In future studies, we plan to obtain this information non invasively with the help of high frequency ultrasonography in anesthetized animals. On the same line, despite repeated attempts, we were not able to obtain consistent results for measurements of the systolic blood pressure along the 4 weeks of the study, hence causal and temporal inference can not be made on the influence played by active study samples for this value.

## Methods

### Materials

Triolein was purchased from Nacalai Tesque (Kyoto, Japan).

### Animals

All animal experiments were approved by the Kindai University Animal Care and Use Committee, and performed according to the Kindai University Animal Experimentation Regulations (Approval number; KAAG-25-001). Six-week-old male Sprague-Dawley rats (SHIMIZU Laboratory Supplies Co., Ltd, Kyoto, Japan) were provided with food (Supplementary Table S1a,b) and water *ad libitum*, in a humidity-controlled room, with a 12-hour light and 12-hour dark cycle. The room temperature was maintained at 25 ± 1 °C.

After habituation for 1 week, the abdominal aorta was ligated over an inserted catheter in all rats to induce AAA. Rats were then orally administered either water (control group), triolein (1145 mg/kg/day), or fish oil (1145 mg/kg/day; purified TG extracted from sardines; Nippon Suisan Kaisha Ltd. Tokyo, Japan) for 4 weeks. Aortic diameters were then measured and the rats sacrificed. When a rat died by AAA rupture, the aortic diameter was measured and the abdominal aorta immediately isolated. Diet composition is shown in Supplementary Table S1a,b. The fatty acid composition of the fish oil is shown in Supplementary Table S1b.

### Induction of hypoperfusion of abdominal aortic wall

The induction of hypoperfusion of the abdominal aortic wall was performed as previously described^10^. First, the infra-renal aorta was exfoliated from the perivascular tissue. Vessels branching from the abdominal aorta were ligated with a 5–0 silk string to block off the blood supply. A plastic catheter (Medikit, Tokyo, Japan), cut short to 5–8 mm in length, was inserted via a small incision adjacent to the renal artery branches and the incision was repaired with a 6–0 monofilament string. The abdominal aorta was ligated with a 5–0 silk string together with the plastic catheter.

### Sample Collection

Rats were anesthetized by intraperitoneal administration of 50 mg/ml pentobarbital (Tokyo Chemical Industry, Tokyo, Japan). The diameter of the abdominal aorta was measured using digital calipers (A&D, Tokyo, Japan). The dilation ratio was calculated according to the following formula: dilation ratio = maximal aneurysm diameter (sac)/non-dilated vascular diameter (neck). An AAA was considered to be formed when the dilation ratio was greater than two. Isolated tissues were fixed in 4% paraformaldehyde (Nacalai Tesque, Kyoto, Japan), soaked in sucrose (10%, 15% and 20%) and embedded in O.C.T. Compound (Sakura Finetek Japan Co., Ltd.). These were stored at −80 °C until required.

### Histological analysis

Isolated aorta cross-sections (10 μm thick) were prepared using a cryostat (CM1850; Leica Microsystems, Wetzlar, Germany) and mounted on glass slides. Aortic walls were stained with Hematoxylin-Eosin (HE) staining, Elastica van Gieson (EVG) staining, PicroSirius Red (PSR) staining, Oil Red O staining and immunohistochemical staining. Quantitative analysis of histological staining was performed using ImageJ software (National Institutes of Health, Bethesda, Maryland, USA). Areas within 100 μm of an adipocyte were defined as ‘around adipocyte’.

### Hematoxylin-Eosin Staining

PFA-fixed tissue sections were placed in hematoxylin for nuclear staining for 10 minutes, and then decolorized in acid alcohol (1% HCl in 70% ethanol). After rinsing in tap water, the sections were stained with eosin for 5 minutes, and then dehydrated in ethanol (80%, 90%, and 100%). Thereafter, the sections were cleared in xylene and covered with a lipid-soluble mounting medium, Entellan^®^ New (Merck KGaA, Germany), and glass cover slips. Quantitative analysis of the thickness of the aortic wall was performed using ImageJ software.

### Elastica van Gieson Staining

PFA-fixed tissue sections were stained in resorcin-fuchsin solution for 30 minutes. After rinsing in tap water, the tissue sections were stained for 10 minutes in a 1:1 mixture of Weigert’s iron hematoxylin solution I (1% hematoxylin in ethanol) and solution II (2% ferric chloride in 0.25% HCl). After rinsing with tap water, the sections were stained with 1% fuchsin solution, diluted 3:20 in van Gieson P solution, for 3 minutes. The tissue sections were dehydrated in ethanol (70%, 90%, and 100%), cleared in xylene, and covered with a lipid-soluble mounting medium and glass cover slips. Elastic lamina was categorized into 4 grades: intact elastic lamina was designated grade 1; deletion of wave form or/and dilution of elastic lamina was designated grade 2; partial disappearance of elastic lamina was designated grade 3; and disappearance of elastic lamina was designated as grade 4, with this being the worst state. EVG stained-sections were then used for the measurement of medial wall thickness.

### PicroSirius Red Staining

PFA-fixed tissue sections were stained in Weigert’s iron hematoxylin solution for 10 minutes. The tissue sections were then decolorized in acid alcohol (1% HCl in 70% ethanol). After rinsing in tap water, the tissue sections were stained in 1% Sirius Red (Waldeck) solution, diluted 1:20 in van Gieson P solution, for 10 minutes. The tissue sections were dehydrated in 100% ethanol, and covered with a lipid-soluble mounting medium and glass cover slips. Quantitative analysis of collagen-positive areas was performed using ImageJ software.

### Oil Red O Staining

PFA-fixed tissue sections were rinsed in 60% isopropanol for 1 minute. The tissue sections were then incubated in Oil Red O solution for 10 minutes. After rinsing in 60% isopropanol for 1 minute, the tissue sections were placed in hematoxylin for nuclear staining for 5 minutes. After rinsing in tap water, slides were covered with an aqueous mounting medium (Nichirei Biosciences, Tokyo, Japan) and glass cover slips.

### Immunohistochemical Staining

PFA-fixed tissue sections were rinsed in phosphate-buffered saline (PBS) with 1% Triton-X100 and then incubated in 10% oxalic acid for 1 hour. For antigen activation, 0.1% trypsin in PBS was added to the tissue sections. Endogenous horseradish peroxidase (HRP) in the tissue sections was blocked using 3% aqueous hydrogen peroxide in methanol for 8 minutes. After washing in PBS, the tissue sections were blocked with Blocking One Histo. The sections were incubated with the appropriate primary antibody overnight at 4 °C. The histological results from the aortic wall were assessed after staining using the following antibodies: rabbit anti-matrix metalloproteinase (MMP) 2 (1:100; Thermo Scientific), goat anti-MMP9 (1:100; Santa Cruz Biotechnology, Inc.), rabbit anti-MCP-1 (1:50; Novus Biologicals), mouse anti-monocytes/macrophages (MAC387) (1:50; Bio-Rad Laboratories), mouse anti-α-smooth muscle actin (1:400; Santa Cruz Biotechnology, Inc.), rabbit anti-CD163 (1:100; Bioss Antibodies). On the following day, the sections were rinsed in PBS, and incubated with the appropriate secondary antibody conjugated to HRP. Slides were developed with DAB (Vector Laboratories, Burlingame, CA, USA), dehydrated in ethanol (80%, 90%, and 100%), cleared in xylene, and covered with a lipid-soluble mounting medium and glass cover slips.

### Human study

The study protocol was reviewed and approved by the Hamamatsu University School of Medicine Ethics Committee of Clinical Research (The Ethic Committee’s approval number is 20372012). All procedures used in this study were carried out in accordance with the Clinical Research Ethics Committee of Hamamatsu University School of Medicine. Written informed consent for use of the aortic tissue samples was obtained from each patient.

We enrolled 30 patients who underwent elective open surgery for the repair of infra-renal AAA at the Division of Vascular Surgery, Hamamatsu University School of Medicine, between April 2008 and March 2012. Aortic diameters were measured pre-operatively by three-dimensional multidetector computed tomography imaging of the AAA. During surgery, longitudinal tissue strips were collected from the aorta, from the nearby distal portion of the bifurcation of the renal artery (neck) to the region of maximal dilation of the aneurysm (sac).

The patients were all male, 58 to 89 years old (mean age 69.8 ± 12.0 years). Tissue samples were preserved in rapid freeze storage until required for analysis with biochemical quantitation.

### Biochemical quantitation

Total lipids were extracted from homogenized tissue. Total cholesterol ester and triglycerides were quantified using colorimetric methods (Test Wako, Wako Pure Chemical Industries, Osaka, Japan).

### Statistical analysis

Values were expressed as mean ± SEM. For between-group comparisons, the Chi-square test or Fisher’s exact test (for situations with small frequencies) was used for categorical variables. Student’s t test for continuous data with Tukey-Kramer test and Mann-Whitney test for scoring data were used. Kaplan–Meier method was used for analysis of survival analysis, and intergroup differences were evaluated by the log-rank test with Holm adjustment for multiple comparisons. A P-value < 0.05 was considered to indicate a statistically significant difference. Statistical analyses were performed using StatView 5.0 software (SAS Institute, Cary, USA) and R 3.2.0 with the EZR package^17^,^18^.

## Additional Information

**How to cite this article**: Kugo, H. *et al.* Adipocyte in vascular wall can induce the rupture of abdominal aortic aneurysm. *Sci. Rep.* **6**, 31268; doi: 10.1038/srep31268 (2016).

## Supplementary Material

Supplementary Information
