# Supplementary material for: Adipocyte in vascular wall can induce the rupture of abdominal aortic aneurysm
Source: Sci Rep. 2016 Aug 8;6:31268. doi: 10.1038/srep31268 (PMC4976321; doi:10.1038/srep31268)

Adipocyte in vascular wall can induce the rupture of abdominal aortic aneurysm

Hirona Kugo[1*], Nobuhiro Zaima[1*], Hiroki Tanaka[2*], Youhei Mouri[1], Kenichi Yanagimoto[3], Kohsuke Hayamizu[3,4], Keisuke Hashimoto[1], Takeshi Sasaki[5], Masaki Sano[6], Tatsuro Yata[6], Tetsumei Urano[2], Mitsutoshi Setou[7,8,9], Naoki Unno[6], Tatsuya Moriyama[1]

[1] Department of Applied Biological Chemistry, Graduate School of Agriculture, Kindai University, 204-3327 Nakamachi, Nara City, Nara 631-8505, Japan

[2]Department of Medical Physiology, Hamamatsu University School of Medicine, Shizuoka, Japan

[3]Human Life Science R&D Center, Nippon Suisan Kaisha, Ltd., Tokyo, Japan

[4] General Health Medical Center, Yokohama University of Pharmacy, Japan

[5] Department of Anatomy and Neuroscience, Hamamatsu University School of Medicine, Japan

[6] Division of Vascular Surgery, Second Department of Surgery, Hamamatsu University School of Medicine, Japan

[7] International Mass Imaging Center Department of Cellular and Molecular Anatomy, and Preeminent Medical Photonics Education & Research Center Department of Systems Molecular Anatomy, Hamamatsu University School of Medicine, 1-20-1 Handayama, Higashi-ku, Hamamatsu, Shizuoka 431-3192, Japan

[8] Department of Anatomy, The university of Hong Kong, 6/F, William MW Mong Block 21 Sassoon Road, Pokfulam, Hong Kong SAR, China

[9] Division of Neural Systematics, National Institute for Physiological Sciences, 38 Nishigonaka Myodaiji, Okazaki, Aichi, 444-8585, Japan

*These authors contributed equally to this work.

*Corresponding author

Animal Study

Nobuhiro Zaima zaima@nara.kindai.ac.jp

Department of Applied Biological Chemistry, Graduate School of Agriculture, Kindai University, 204-3327 Nakamachi, Nara City, Nara 631-8505, Japan

Clinical Study

Naoki Unno unno@hama-med.ac.jp

Division of Vascular Surgery, Second Department of Surgery, Hamamatsu University School of Medicine, Japan

Keywords: abdominal aortic aneurysm, rupture, adipocyte, triglyceride, fish oil

Supplementary Information

Supplementary Table S1 Diet composition
(a) Diet composition and (b) fatty acid composition of coconut oil and fish oil.

Supplementary Fig. S1 Ruptured AAA, pictured before washing away blood.

Supplementary Fig. S2 Weight change and serum parameters
(a) Initial body weight of rats in the non-ruptured and ruptured groups. (b) Body weight gain of rats in the non-ruptured and ruptured groups. Serum triglyceride (TG) levels (c) and total cholesterol levels (d) of rats in the non-ruptured and ruptured group. Data are the mean ± s.e.m. Non-ruptured (n = 5), ruptured (n = 5).

Supplementary Fig. S3 Vascular wall thickness and elastin degradation score
(a-d) Representative images of HE staining (scale bar = 200 μm). (e-h) Representative images of EVG staining (scale bar = 100 μm). (i) Quantification of vascular wall thickness in the non-ruptured and ruptured groups. (j) Elastin degradation scores in the non-ruptured and ruptured groups. Data are the mean ± s.e.m. Non-ruptured (n = 5), ruptured (n = 5). Values with different letters are significantly different ($P < 0.05$).

Supplementary Fig. S4 Thickness of medial wall
(a-d) Representative images of immunostaining for α-smooth muscle actin (scale bar = 100 μm). (e) Quantification of α-smooth muscle actin-positive areas of vascular wall. (f) Quantitative analysis of medial wall thickness in the non-ruptured and ruptured groups. Values with different letters are significantly different ($P < 0.05$).

Supplementary Fig. S5 Observation of adipocyte and PPARγ staining
Representative electron microscopic image of the vascular wall of the AAA neck (a) and sac (b) from a model rat. Representative optical images of the neck (c) and sac (d) (Scale bars = 200 μm). Representative images of PPARγ staining of neck (e) and sac (f) (Scale bars = 10 μm). Red: PPARγ. Blue: nucleus.

Supplementary Fig. S6 Immunohistochemical staining for CD163
(a-e) Representative images of immunostaining for CD163 (scale bar = 50 μm). N.D.: not detected. (f) Quantification of CD163-positive areas of the vascular wall. Values with different letters are significantly different ($P < 0.05$).

Supplementary Fig. S7 Weight change and serum parameters

(a) Body weight change of rats in the triolein and fish oil groups. Serum triglyceride (TG) levels (b) and total cholesterol levels (c) in the control, triolein and fish oil groups. Data are the mean ± s.e.m. Control group (n=10), triolein group (n = 13), fish oil group (n = 12). * $P< 0.05$ versus triolein group.

Supplementary Fig. S8 Thickness of vascular wall

AAA sac areas from the three experimental groups were divided into two groups: those with areas without adipocytes (-) (b, e and h) and those with adipocytes (+) (c, f and i). (a-i) Representative images of HE staining of the control group (a-c), triolein group (d-f) and fish oil group (g-i). (a, b, d, e, g, h: scale bar = 100 μm; c, f, i: scale bar = 400 μm). (j) Quantitative analysis of vascular wall thickness of the control, triolein and fish oil groups. Data are the mean ± s.e.m. Control group (n=9), triolein group (n = 10), fish oil group (n = 8). Values with different letters are significantly different ($P< 0.05$).

Supplementary Fig. S9 Elastin degradation score and collagen-positive area

AAA sac areas from the three experimental groups were divided into two groups: those with areas without adipocytes (-) (b, e, h, l, o and r) and those with adipocytes (+) (c, f, j, i, m, p and s). (a-i) Representative images of EVG staining of the control group (a-c), triolein group (d-f) and fish oil group (g-i) (a, b, d, e, g, h: scale bar = 100 μm; c, f, i: scale bar = 200μm). (j) Elastin degradation scores in the control, triolein and fish oil groups. (k-s) Representative images of PSR staining of the control group (k-m), triolein group (n-p) and fish oil group (q-s) (k, n, q: Scale bar = 100 μm, l, o, r: scale bar = 400 μm, m, p, s: scale bar = 50 μm). (t) Quantification of the collagen-positive areas of the vascular wall. Data are the mean ± s.e.m. Control group (n=9), triolein group (n = 10), fish oil group (n = 8). Values with different letters are significantly different ($P< 0.05$).

Supplementary Fig. S10 Thickness of medial wall

(a-f) Representative images of immunostaining for α-smooth muscle actin (scale bar = 100 μm). (g) Quantification of α-smooth muscle actin-positive areas of the vascular wall. (h) Quantitative analysis of medial wall thickness in the control, triolein and fish oil groups. Values with different letters are significantly different ($P< 0.05$).

Supplementary Fig. S11 Immunohistochemical staining for MCP-1 and $MAC387^{+}$ monocytes/macrophages

AAA sac areas from the three experimental groups were divided into two groups: those with areas without adipocytes (-) (b, e, h, l, o and r) and those with adipocytes (+)(c, f, j, i, m, p and s). (a-i) Representative images of immunostaining for MCP-1 (scale bar = 50 μm). (j) Quantification of MCP-1-positive areas of the vascular wall. (k-s) Representative images of immunostaining for MAC387$^+$ monocytes/macrophages (scale bar = 50 μm). (t) Quantification of MAC387$^+$ monocytes/macrophage-positive areas of the vascular wall. Data are the mean ± s.e.m. Control group (n=9), triolein group (n = 10), fish oil group (n = 8). Values with different letters are significantly different ($P < 0.05$).

Supplementary Fig S12 Relationships between serum lipids, tissue lipids, BMI and aortic diameter in human AAA samples.

The amount of triglyceride (TG) in aortas versus serum TG levels (a), serum total cholesterol levels (b) and body mass index (BMI) (c). The total cholesterol amount in aortas versus serum TG levels (d), serum total cholesterol levels (e) and BMI (f). Aortic diameter versus serum TG levels (g), serum total cholesterol levels (h) and BMI (i). Aortas were divided into two groups: 1) intima and media, and 2) adventitia.

Supplementary Table S1 Diet composition

(a)

Diet composition	(%)
choline chloride	0.25
cystine	0.3
AIN-93 vitamin mix	1
AIN-93G mineral mix	3.5
cellulose	5
sucrose	10
casein	20
cornstarch	55.75
coconut oil	4.2
	100

(b)

Fatty acid	coconut oil (%)	fish oil (%)
8 : 0	7.1	-
10 : 0	7.6	-
12 : 0	27.6	-
14 : 0	19.8	5.2
16 : 0	14.9	6.6
16 : 1	-	9.3
16 : 2	-	1.7
16 : 3	-	2.8
16 : 4	-	4.8
18 : 0	6.6	0.5
18 : 1	13.3	9.6
18 : 2 n-6	3.1	1.4
18 : 3 n-3	-	0.9
18 : 4 n-3	-	5.1
20 : 4 n-6	-	1.3
20 : 4 n-3	-	1.1
20 : 5 n-3	-	30.8
22 : 5 n-6	-	0.3
22 : 5 n-3	-	2.9
22 : 6 n-3	-	15.7

Supplementary Fig. S1 Ruptured AAA before washing blood.

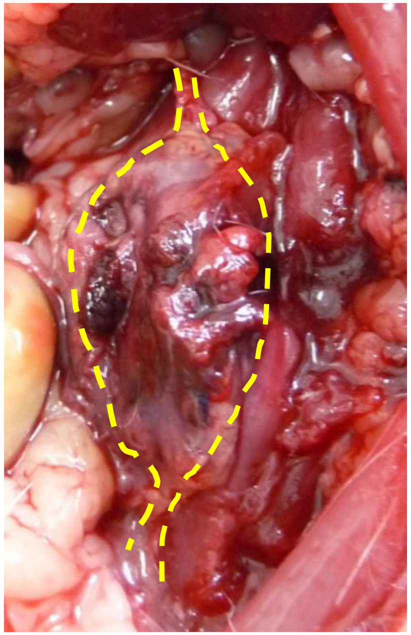

Supplementary Fig. S2 Weight change and serum parameters

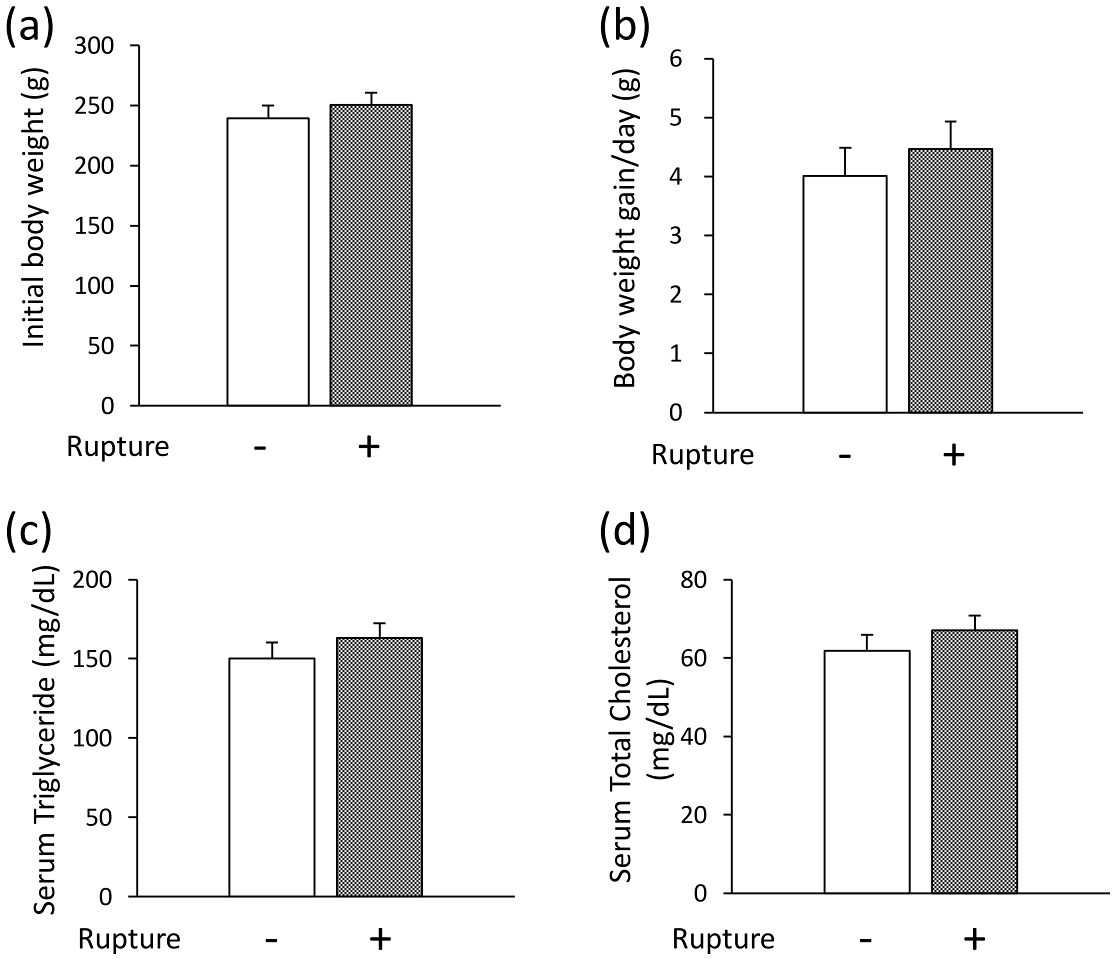

Supplementary Fig. S3 Vascular wall thickness and elastin degradation score

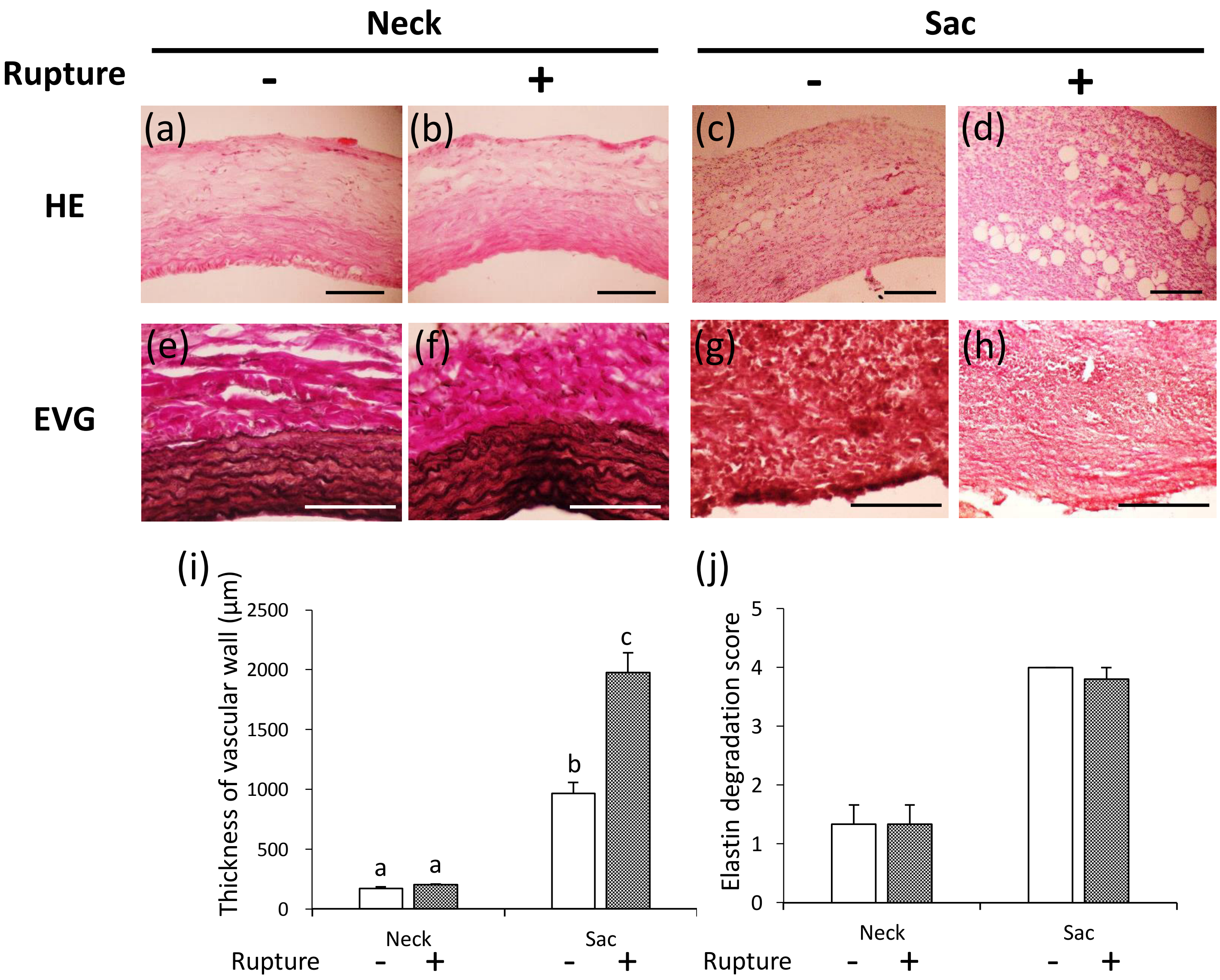

Supplementary Fig. S4 Thickness of medial wall

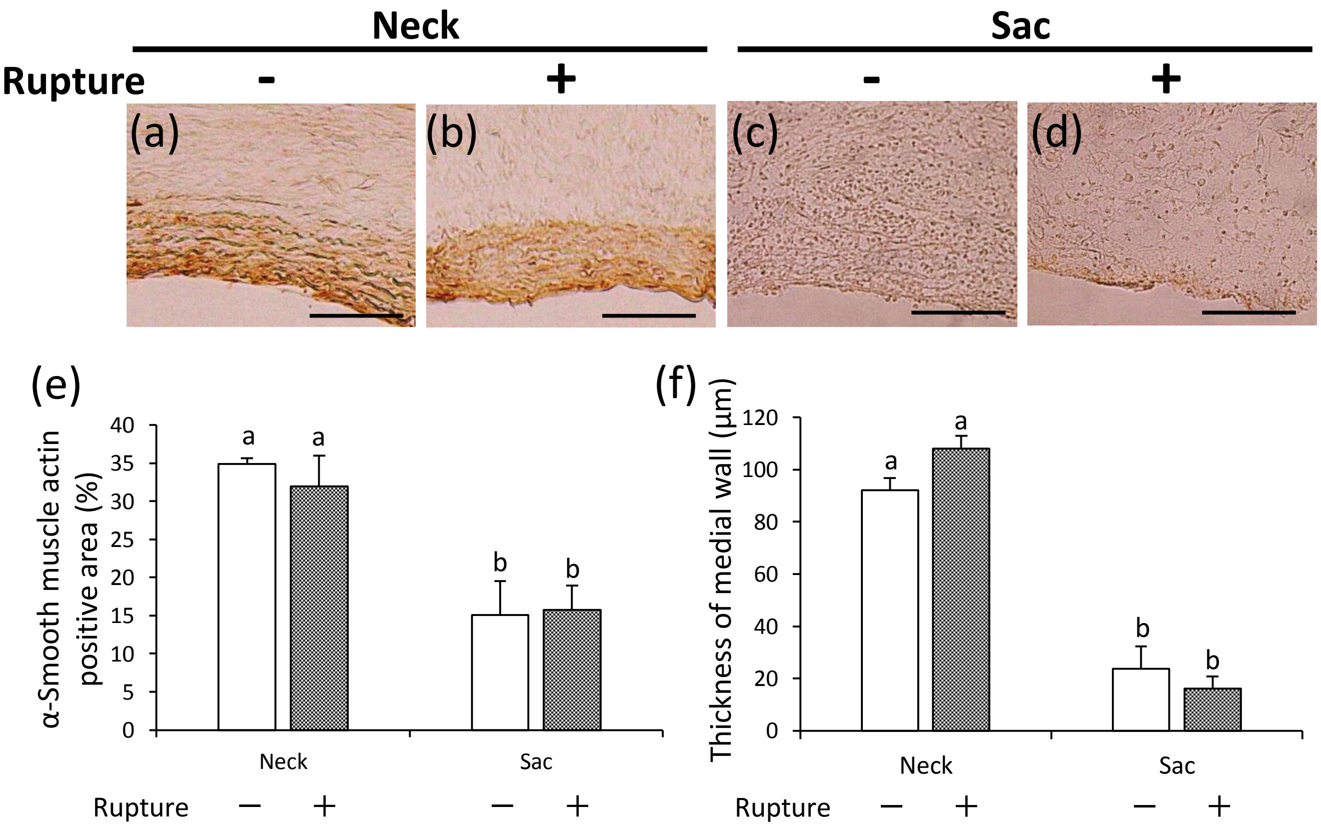

Supplementary Fig. S5 Observation of Adipocyte and PPARγ staining

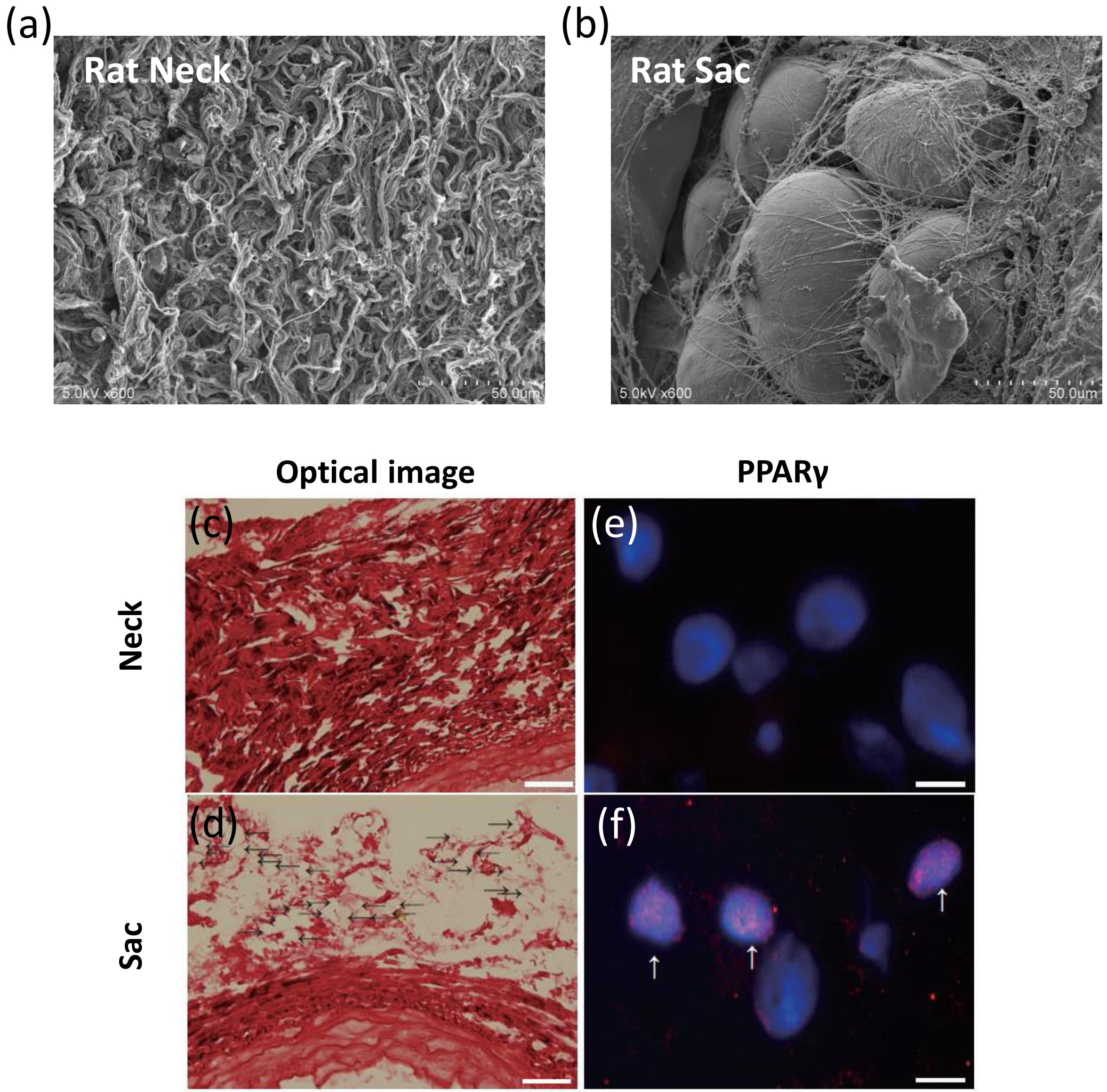

Supplementary Fig. S6 Immunohistochemical staining for CD163

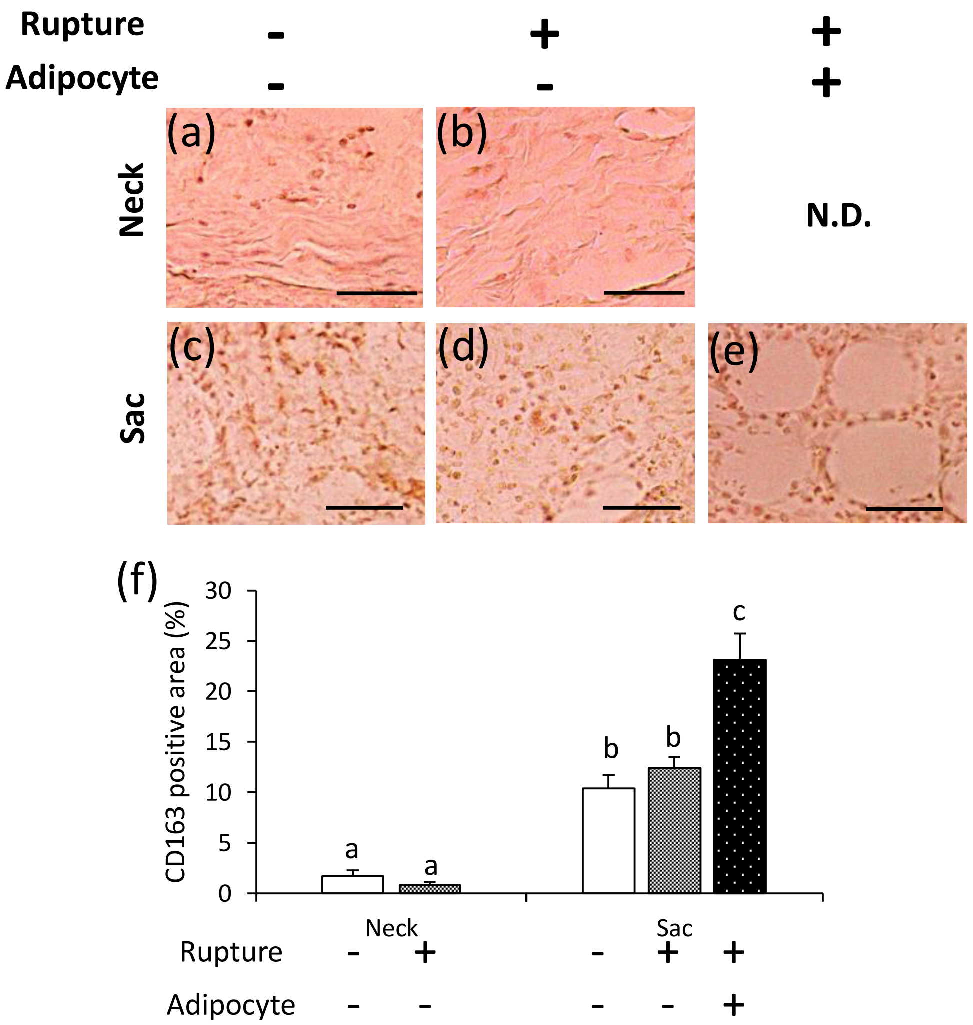

Supplementary Fig. S7 Weight change and serum parameters

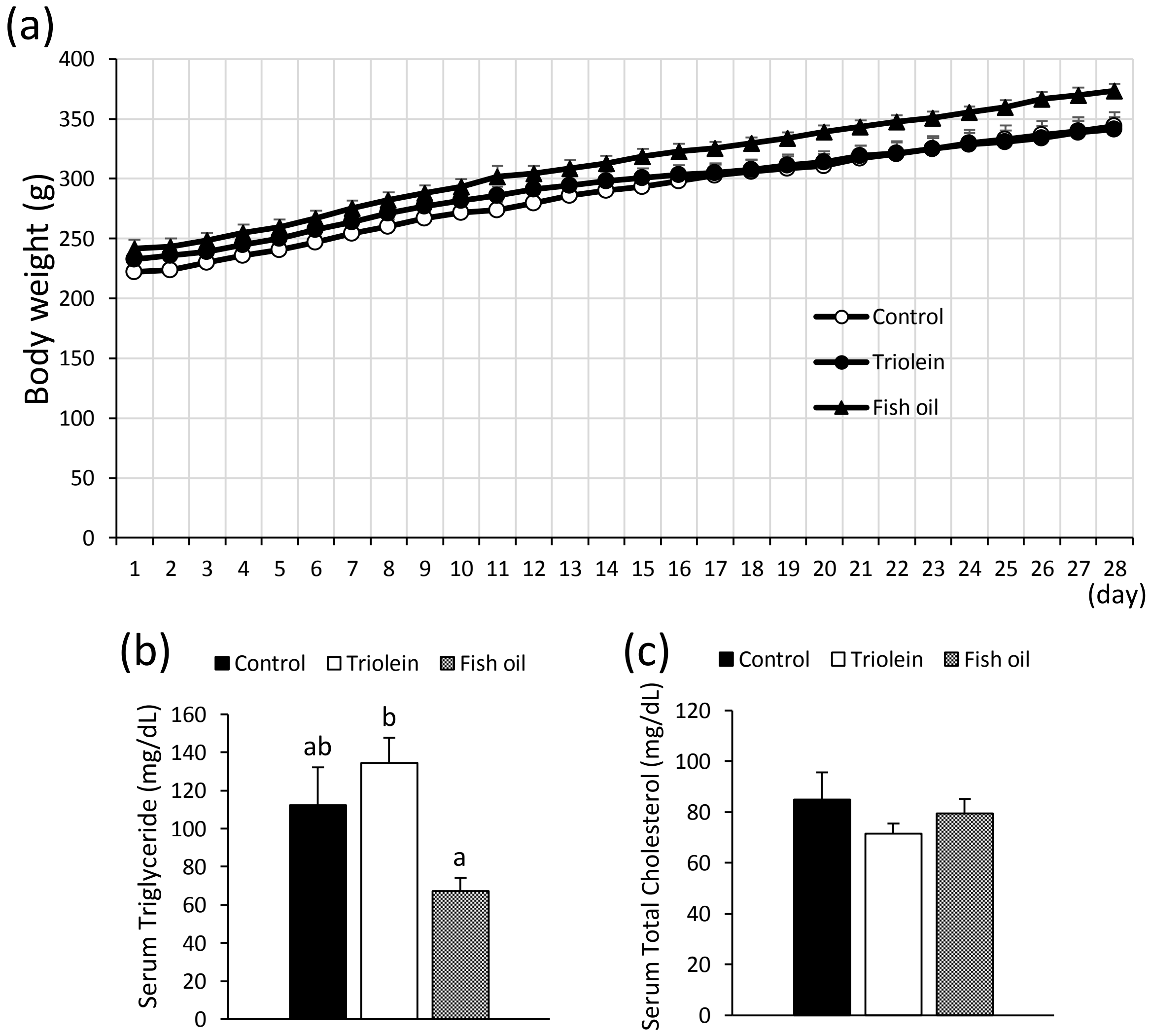

Supplementary Fig. S8 Thickness of vascular wall

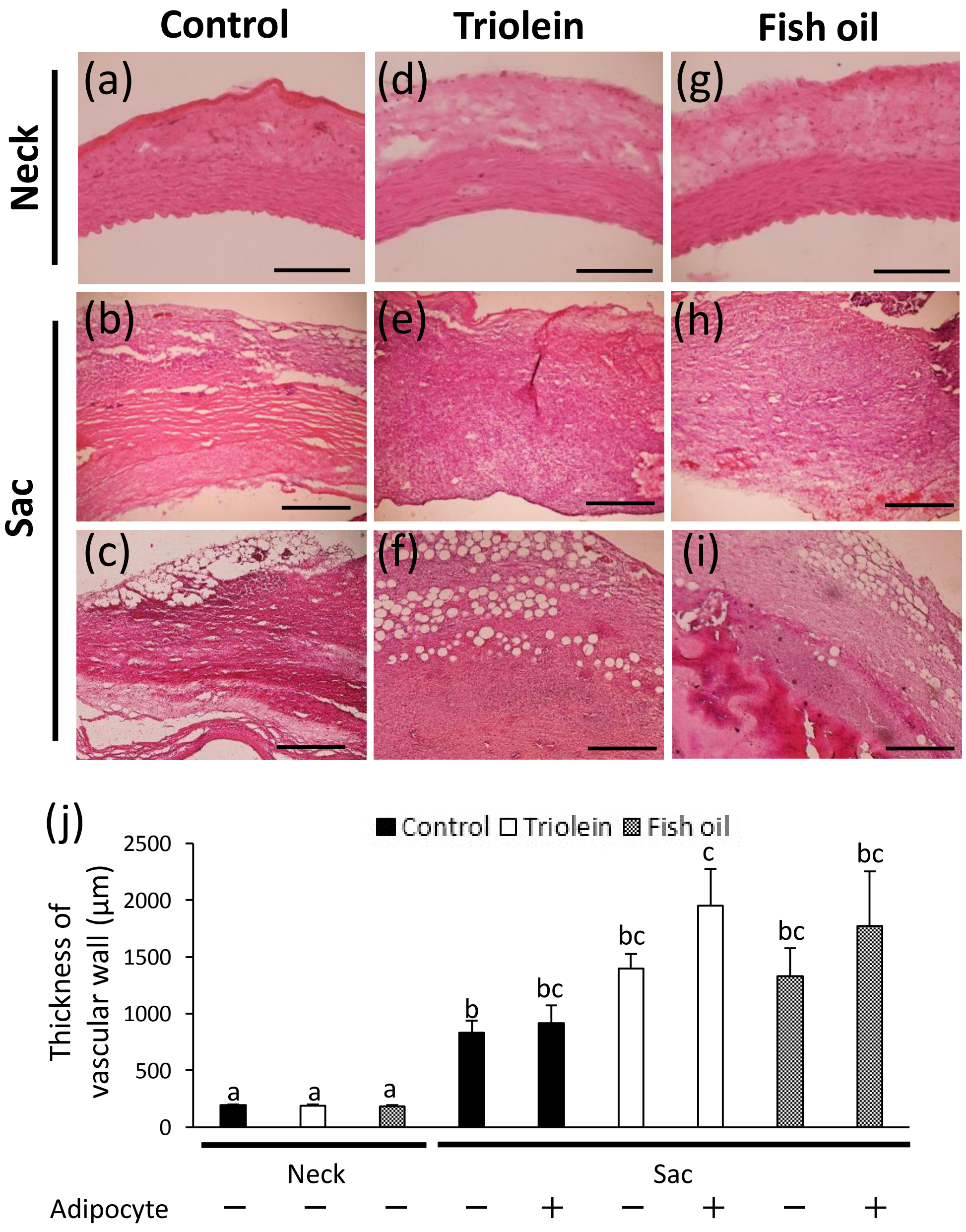

Supplementary Fig. S9 Elastin degradation score and collagen-positive area

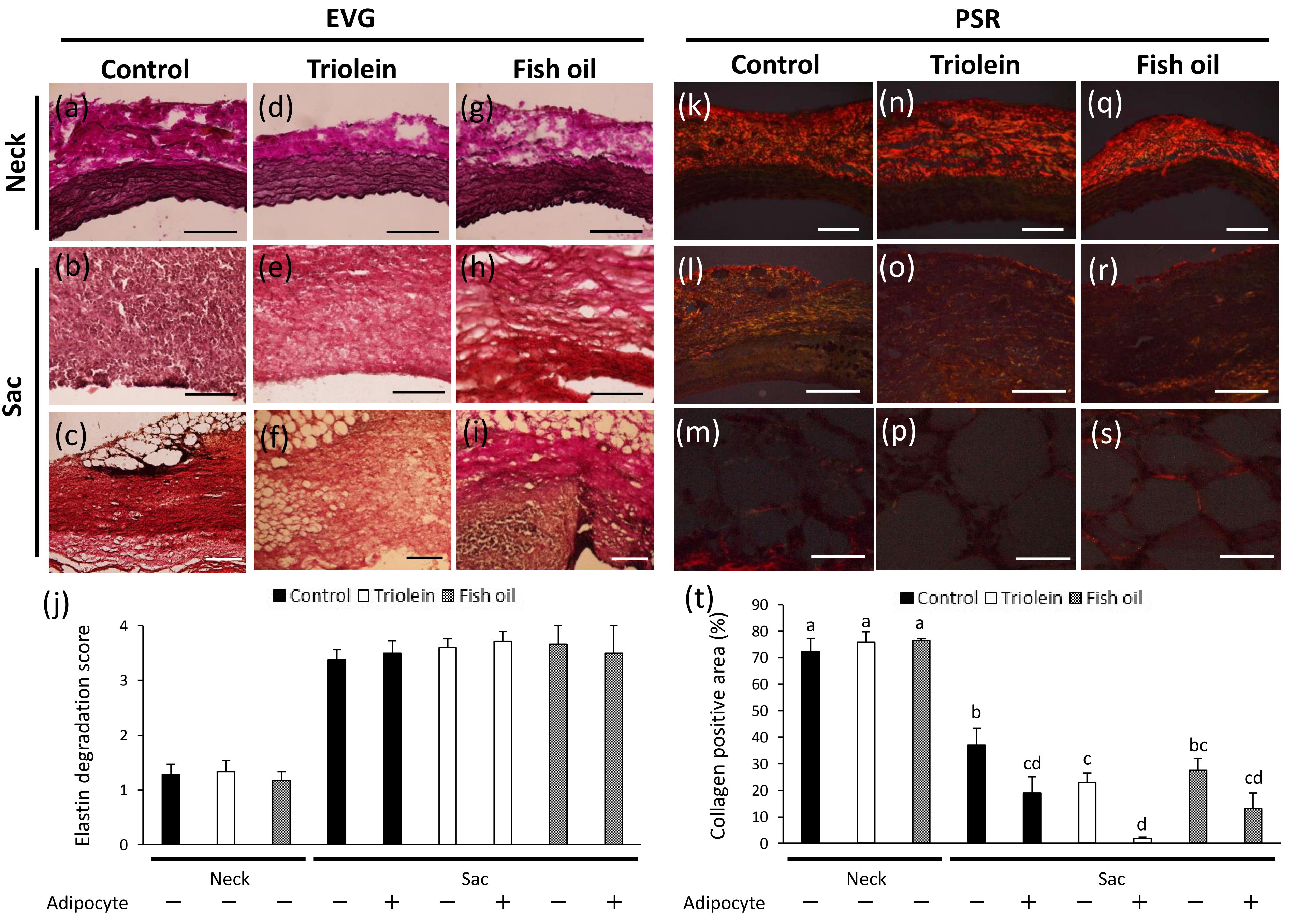

Supplementary Fig. S10 Thickness of medial wall

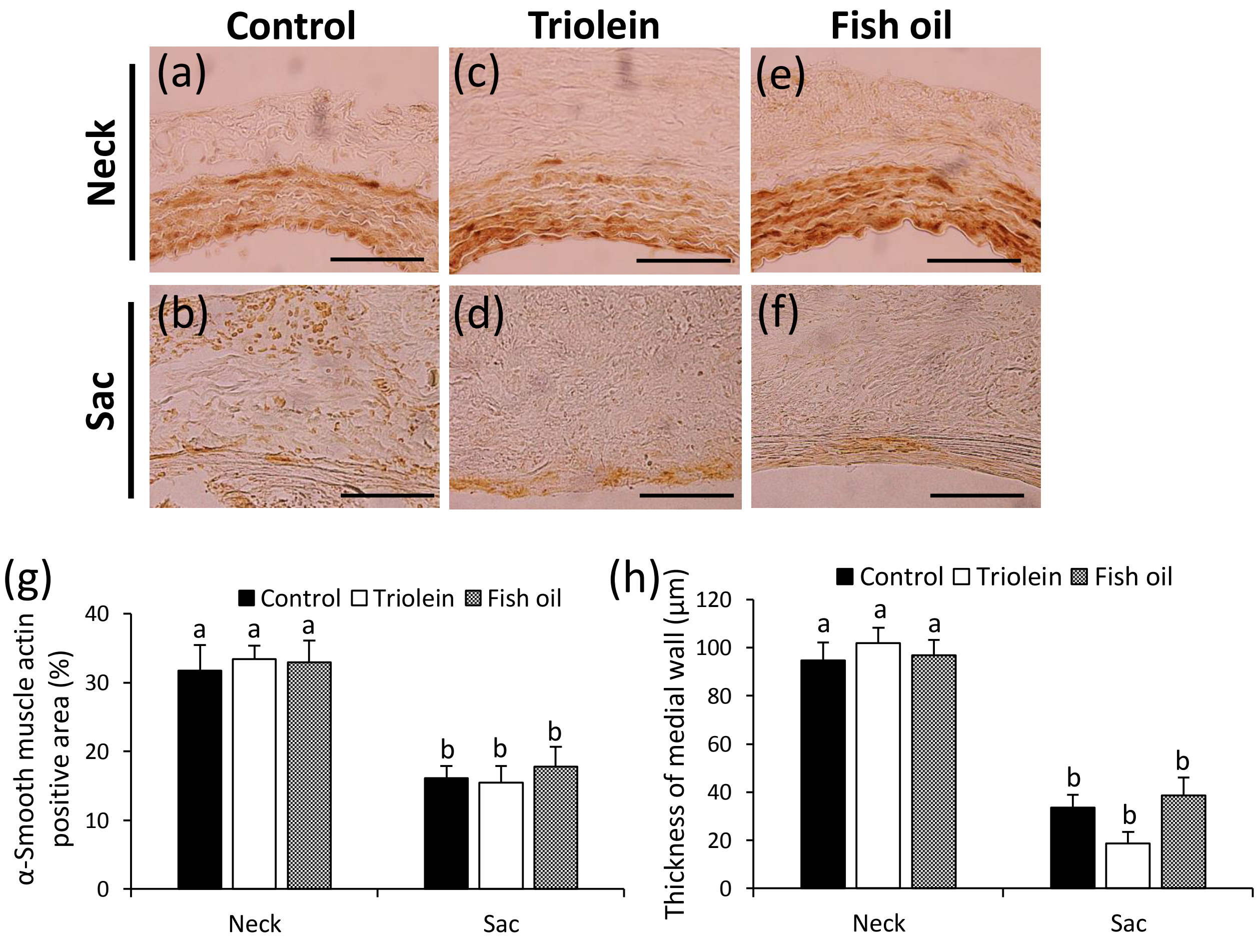

Supplementary Fig. S11 Immunohistochemical staining for MCP-1 and MAC387+ monocytes/macrophages

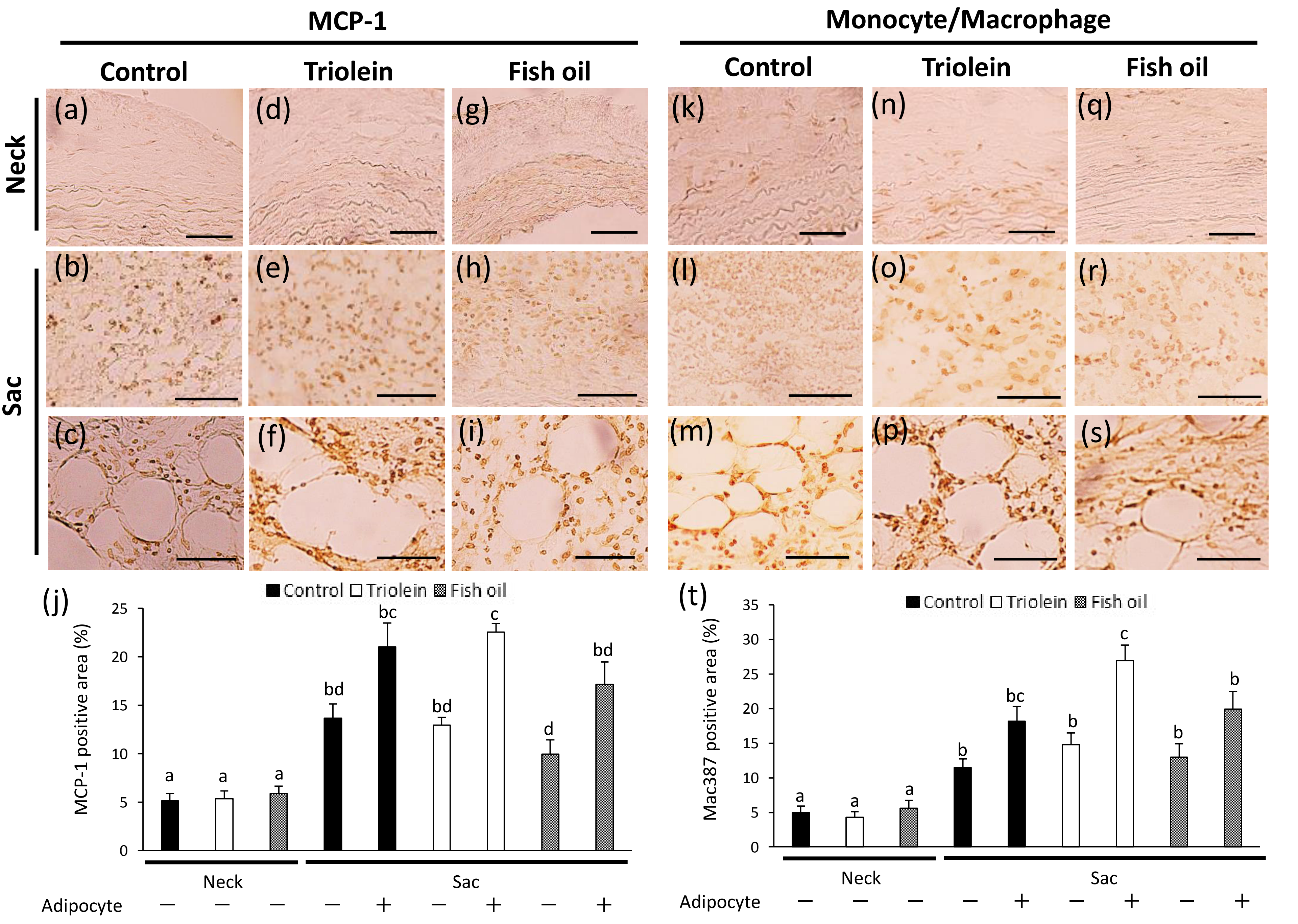

Supplementary Fig. S12 Relation between serum lipids, tissue lipids, BMI and aortic diameter

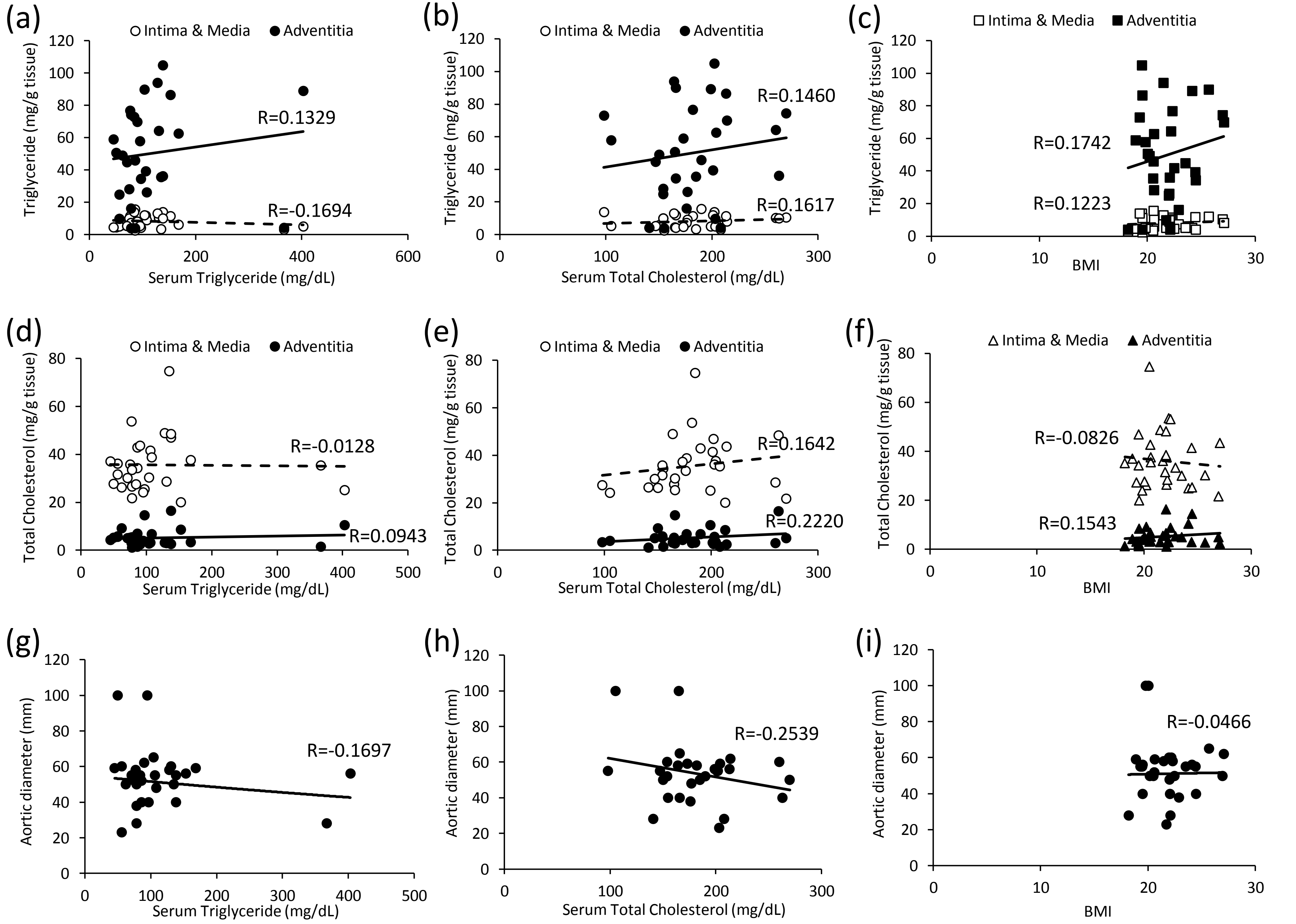

Supplement: Supplementary Information [file srep31268-s1.pdf]
